# Medical Education During COVID-19 Pandemic: A Comparative Effectiveness Study of Face-to-Face Traditional Learning Versus Online Digital Education of Basic Sciences for Medical Students

**DOI:** 10.7759/cureus.35837

**Published:** 2023-03-06

**Authors:** Adekunle E Omole, Maria Elena Villamil, Hassan Amiralli

**Affiliations:** 1 Anatomical Sciences, American University of Antigua, College of Medicine, Saint John, ATG; 2 Clinical Medicine, American University of Antigua, College of Medicine, Saint John, ATG

**Keywords:** traditional offline learning, medical education, online education, face-to-face teaching, coronavirus pandemic

## Abstract

Background: During the coronavirus disease 2019 (COVID-19) pandemic, internet-based learning modalities and online classes became a tool for the continuous learning process for medical students. The aim of this study was to compare medical student performance in both online versus offline instructional methods.

Methods: The study was conducted on 213 medical students of the basic science program at the American University of Antigua, College of Medicine (AUACOM), who completed the four semesters consecutively between Spring 2018 and Fall 2020. Two cohorts of students were considered in the study: cohort 1 (those who completed years 1 and 2 using traditional offline teaching modality) and cohort 2 (those who completed year 1 offline and year 2 online). The years 1 and 2 National Board of Medical Examiners (NBME) summative assessment scores of the students were used to determine which instructional modality generated better student performance outcomes for the two groups. Additionally, we evaluated the score variabilities between genders to determine if teaching modality had an impact on a specific group. All statistical comparisons were done using two-tailed *t*-tests.

Results: The study involves 213 students (112 in cohort 1, 101 in cohort 2). There was no significant difference in student performance between offline and online learners overall (74 ± 2.3 vs. 73 ± 1.3; p = 0.537) or with respect to gender (73 ± 3.8 vs. 73 ± 3.0; p = 0.709).

Conclusion: In this comparative effectiveness study of traditional offline education versus online instructional modality, we observed no statistical difference in student performance evaluated with NBME summative assessment scores. Online classes were well-accepted by our students. These data show a significant and promising potential for the future of medical education using online teaching modalities. Remote online teaching could be used again in the future without detriment to student education if face-to-face (F2F) learning is not possible.

## Introduction

The coronavirus disease (COVID-19) was first detected in December 2019 in Wuhan, China and within months, it spreads rapidly across the globe. It was declared a pandemic by the World Health Organization in March 2020 [1]. This declaration resulted in an unprecedented public health response as governments across the world began to implement a wide range of measures to control and prevent the transmission of the disease. These measures included social distancing, banning of public gatherings, nationwide lockdown, and closure of businesses including educational and public institutions. These measures had a profound impact on the population, the economy and in particular the education system.

With the advancement of digital technology, digital online education became a common teaching method as an alternative to traditional in class education during the COVID-19 pandemic. Online education offers more flexibility since it is not time and place-bound. However, there is a consensus that medical education requires in-person didactic sessions, skill-based and a team-based approach [2]. Thus, the COVID-19 pandemic presents a challenge for medical education administrators, who were forced to shift from offline learning or traditional face-to-face (F2F) teaching to online digital learning [3]. Most institutions, including the American University of Antigua, College of Medicine (AUACOM), had to make the paradigm shift from offline to online teaching within several days of the COVID-19 pandemic declaration to have a continuum in students’ education.

Many medical educational institutions have reported conflicting outcomes in their students' experiences using the online learning modality during COVID-19 pandemic [4-8]. Overall, few studies have investigated the effectiveness of online learning modality on medical students' education. Review of the literature revealed that no comparative analysis study of medical student performance in an online vs. traditional F2F instructional method has been carried out in the Caribbean region. The aim of our study was to compare the outcome of our students’ summative assessments in both online versus offline medical education. Additionally, we examined score variabilities between genders to determine if instructional modality had an impact on any group. The results of this research are intended to provide us with information on which teaching modality is significantly more effective than the other, and to inform educators, administrators, and policy-makers on the outcome of these new changes in medical education.

## Materials and methods

The data we analyzed for this study is anonymous secondary data of National Board of Medical Examiners (NBME) scores retrieved from the AUACOM examination center database. The study sample consisted of 213 basic science students at AUACOM who completed four semesters consecutively between Spring 2018 and Fall 2020 (S2018-F2020). Our two-year basic science curriculum includes four semesters, the spring and fall semesters of years 1 and 2, respectively. In order to successfully complete the four semesters of the two-year basic science program consecutively, a student must achieve a yearly total average score of $\geq$ 70% in their Customized Assessment Services (CAS) exams. CAS exams are provided by the NBME. Students who have repeated any of the semesters/years were excluded from the study for the sake of consistency in skill/ability. The final average score of the participant’s year 1 and year 2 grades of CAS NBME exams serves as the main comparative factor in assessing performance differences between online and offline F2F instruction.

The 213 total participants consisted of two cohorts of students. Cohort 1 students attended the basic science program from S2018-F2019 and completed both years 1 and 2 using the traditional offline teaching modality. Cohort 2 students attended the basic science program from S2019-F2020 and completed year 1 using the traditional offline teaching modality while year 2 was completed via online mode taking into consideration the COVID-19 operational guidelines. Cohort 1 and 2 students were 112 (48 males and 64 females) and 101 (48 males and 53 females), respectively. None of the two cohorts consisted of an online-only group. This disparity was considered a limitation of our study. Due to the nature of the composition of the two cohorts, we proposed four research questions for our study.

Research Question 1: Are there significant differences in the academic performance in year 1 between cohort 1 (offline) and cohort 2 (offline) students enrolled in our basic science program?

Research Question 2: Are there significant differences in the academic performance in year 2 between cohort 1 (offline) and cohort 2 (online) students enrolled in our basic science program?

Research Question 3: Are there significant differences in the academic performance when the overall 2-year grade (year 1 + year 2) is compared between cohort 1 (offline) and cohort 2 (offline and online) students enrolled in our basic science program?

Research Question 4: Are there significant differences in the academic performance when the overall 2-year grade (year 1 + year 2) is compared within cohort 1 (offline) and cohort 2 (offline and online) students enrolled in our basic science program with respect to gender?

All statistical analyses were performed using STATA 16 (for windows) to assess for possible associations and statistical significance. The differences between groups were assessed using a t-test. Statistical hypothesis tests with p-values $\leq$ 0.05 were considered significant. Values are presented as mean and standard deviation (SD).

## Results

The NBME CAS Exam yearly average score results for cohort 1 (those who completed year 1 and 2 by F2F learning modality) were compared with results for cohort 2 (those who completed year 1 by F2F learning modality and year 2 by remote online learning modality).

Research question 1

The first research question investigated if there was a statistically significant difference in academic performance in year 1 between cohort 1 (offline) and cohort 2 (offline). This subsection (year 1) of the two cohorts had similar curriculum and both are offline. The aim was to see from the onset if under the same teaching conditions, both cohorts were similar or different.

Table 1 shows us the mean and SD for year 1 grade distribution for the two cohorts. The year 1 mean score for both cohorts is similar with cohort 1 scoring a 74 (SD 1.3) and cohort 2 scoring a 73 (SD 4.8). The correlating p-values for comparisons between year 1 grade between the two cohorts are 0.518. This p-value is greater than our p-value significance level of 0.05. Thus, there is no evidence of a statistically significant difference between the two cohorts in terms of performance scores for year 1. This indicates to us that under the same teaching conditions, the year 1 performance were both similar for the two cohorts considered.

**Table 1:** Year 1 average exam results for each cohort of students (N=213)

Cohort	Study modality	Number	Year 1		t-test Year 1
			Mean Score (%)	SD	p-value
1 (Spring 2018-Fall 2018)	Year 1 on campus (Offline)	112	74	1.3	0.518
2 (Spring 2019-Fall 2019)	Year 1 on campus (Offline)	101	73	4.8	

Research question 2

The second research question was to determine if there was a statistically significant difference in academic performance in year 2 between cohort 1 (offline) and cohort 2 (online). Does online and offline student academic performance vary under the same curriculum?

Table 2 shows us the mean and SD for year 2 grades for the two cohorts. The year 2 mean score for both cohorts is similar with cohort 1 scoring a 74 (SD 2.3) and cohort 2 scoring a 73 (SD 1.3). The SDs were fairly similar in both cohorts. The correlating p-values for comparisons between year 2 grade between the two cohorts is 0.537. This p-value is greater than our p-value significance level of 0.05. Thus, there was no evidence to reject the null hypothesis and there was not a statistically significant difference between the two cohorts in terms of year 2 performance scores (online and F2F offline learners). This indicated to us that the change to online teaching modality by cohort 2 students did not seem to affect their year 2 CAS results, compared to cohort 1 offline students.

**Table 2:** Year 2 average exam results for each cohort of students

Cohort	Study modality	Number	Year 2		t-test Year 2
			Mean Score (%)	SD	p-value
1 (Spring 2019-Fall 2019)	Year 2 on campus (Offline)	112	74	2.3	0.537
2 (Spring 2020-Fall 2020)	Year 2 remotely (Online)	101	73	1.3	

Research question 3

The third research question was posed to determine if there was a difference between cohort 1 (offline) and cohort 2 (offline and online) students when their overall two-year grade (year 1 + year 2) are compared.

Table 3 shows a p-value of 0.342 when overall 2-year grade were compared between cohort 1 and cohort 2. The t-test analysis shows no evidence of statistically significant difference, suggesting that the change by cohort 2 students to online teaching modality during the pandemic did not seem to affect their overall 2-year NBME CAS results, compared to those offline for 2 years.

**Table 3:** Year 1 + Year 2 average exam results for each cohort of students

Cohort	Study modality	Number	Year 1		t-test Y1	Year 2		t-test Y2	t-test Y1 and Y2
			Mean Score (%)	SD	p-value	Mean Score (%)	SD	p-value	p-value
1 (Spring 2018-Fall 2019)	Year 1 and 2 on campus	112	74	1.3	0.518	74	2.3	0.537	0.342
2 (Spring 2019-Fall 2020)	Year 1 on campus, Year 2 remotely	101	73	4.8		73	1.3		

Research question 4

The fourth research question tried to determine if there was a difference within cohort 1 (offline) and cohort 2 (offline and online) students when their overall two-year grade (year 1 + year 2) are compared with respect to gender.

Table 4 reveals the NBME CAS exam results analysis by gender, for both cohorts. The overall two-year grade in cohort 1, showed no evidence of a statistically significant difference between women and men (Mean $\pm$ SD: 73 $\pm$ 2.7 and 76 $\pm$ 2.5, respectively, p =0.064). For cohort 2 students, analysis reveals no statistically significant difference between women and men (Mean $\pm$ SD: 73 $\pm$ 3.8 and 73 $\pm$ 3.0, respectively, p=0.709). Thus, there is no statistically significant difference between men and women in both cohorts with regards to academic performance.

**Table 4:** Year 1 + Year 2 average exam results’ comparison by gender

Cohort	Study modality	Gender	Number	Year 1 and Year 2		t-test Y1 and Y2
				Mean Score (%)	SD	p-value
1 (Spring 2018-Fall 2019)	Year 1 and 2 on campus	Women	64	73	2.7	0.064
		Men	48	76	2.5	
2 (Spring 2019-Fall 2020)	Year 1 on campus, Year 2 remotely	Women	53	73	3.8	0.709
		Men	48	73	3.0	

Summary of the results

In summary, the two-tailed t-tests analysis showed no significant differences in student performance between online and face-to-face learners overall or with respect to gender.

## Discussion

The results of this comparative effectiveness study of two evidence-based approaches to curricular delivery show that there is no significant difference in academic performance between online and traditional F2F classroom students with respect to teaching modality or gender in the basic science preclinical program for medical students in our institution. These data provide supporting evidence for online learning modality as a means to deliver a preclinical curriculum for undergraduate medical education. To the best of our knowledge, this is the first study of its kind in a Caribbean medical school. Our study can also be considered innovative because generally, in the literature, there is sparse data on pedagogical modality comparisons in the medical students population.

In a 2019 meta-analysis comparing online versus offline learning in undergraduate medical education, online learning was shown to be either equal to or superior to face-to-face traditional learning modality [9]. The meta-analysis involved several studies from key journals of medical education during the period from 2000 to 2017. The authors concluded that online learning is at least as effective as offline learning, with the former having advantage to enhance undergraduate knowledge and skills. The authors also postulated that online learning could promote increased self-directed learning. We draw a similar conclusion in our study. However, this conclusion does not suggest that online learning will be an effective teaching modality for every student in every learning setting. The effectiveness of online learning is influenced by the characteristics of students themselves, such as learning style, level of satisfaction, level of engagement, and attitude [10-13]. Indeed, during the transition of our curriculum from offline to online modality due to the COVID-19 pandemic, we observed that in general, students who apply themselves diligently tend to be successful irrespective of the format of learning. The format of online learning used by medical educators is another strong determinant of the learning outcome. A strong curriculum and good pedagogical practices form the foundation of online teaching [14,15]. Effective online teaching modality requires teaching skills that may vary from those medical educators are used to during traditional F2F teaching [16]. Educators must not only be aware of their student's needs, but they must also know how this need can be met effectively. In order to meet this need during the time COVID-19 pandemic, all medical educators in our institution underwent extensive faculty development training on “good online teaching practices” that include instructions on ensuring active engagement, promoting self-directed learning, timely feedback, management of online teaching platforms, etc. [15].

Another study evaluated the similarities and differences in student performance and satisfaction before and after the implementation of online instruction of a 4-week introductory pathology course by first-year medical students [17]. Assessments which included quizzes, and practical and final exams were compared between the offline and online learning modalities. Student performance and course satisfaction generally improved with remote instruction. The researchers concluded that the transition from offline to remote instruction was not associated with a degradation in student performance and in fact, may be accompanied by improvement. They stated that “students were overall very pleased with the course delivered remotely, which scored higher on their evaluations compared to the previous year” [17].

Other studies evaluated students' preferences (but not efficacy) for online vs offline learning while others investigated the general impact of the COVID-19 pandemic on medical education. A cross-sectional study, conducted from February 2021 to April 2021 via social media on second, third, and fourth-year medical students of six Caribbean medical schools (including our institution, AUACOM), revealed that over 60% of the students preferred offline F2F teaching modality over online learning [18]. Most students reported being less time efficient and paying less attention during online lectures. Many reported a delay in their examinations or research projects due to the COVID-19 pandemic lockdown. Most students ranked 10/10 on anxiety and depression scores during the lockdown. This study reminds us that the transition from offline to online teaching modality during the pandemic was not at all a smooth experience for many students. Our students had to eventually adapt to online education. The student advising services of our institution had to work intensively to address many stressors reported by students. Online education lacks one-to-one or face-to-face interaction between the student and the instructor, thus, possibly lacking more personal support. Nevertheless, online education when compared to F2F education is cost-effective and offers more access and flexibility since it is not time and place bound.

Today’s generation of modern learners is technology and digital-savvy with high rates of satisfaction with online learning [19-21]. With technological advancement, it was possible to conduct classes remotely for medical education which could not have been possible if the COVID-19 pandemic had struck few years back. The authors hereby subscribe to the emerging concept of blended/hybrid learning model that integrates online learning with traditional face-to-face learning [22]. This heterogenous approach to medical education will meet the interactivity, connectivity and flexibility expectations of today’s modern learners, allowing us to maximize the benefits of online and face-to-face teaching.

Limitation

Our study has some limitation which must be considered. The study was conducted in a single medical school which limits the generalizability of our findings to other medical institution in other environments. Our study only use the NBME scores of the students to determine the instructional modality that generated better student performance outcome. Theoretical and practical knowledge assessments are both parts of medical education. Practical knowledge assessment using Objective Structured Clinical Examination (OSCE) and other forms of clinical examination were not considered in our study. Additionally, our study did not take into consideration the needs of cohort 2 students and their period of adjustment as they transition from offline to online learning modality. There exists the possibility that these factors may have affected their overall performance. It would be interesting to follow up on cohort 2 groups and compare the performance of an online-only group with an offline-only cohort 1 group. As we gradually transition back into offline learning, there is need for a large multi-centered comparative effectiveness study of offline vs online learning in medical education. Multicenter investigation could be invaluable to analyze the outcome of hybrid or blended teaching methods in medical education. Other variables such as age, family commitments, financial circumstances, access to resources like electricity and internet, that could have had an effect on performance during the pandemic, were not available in this study.

## Conclusions

In the context of the COVID-19 pandemic, remote delivery of the preclinical course was an effective and generally acceptable option for our medical students with no evidence of detriment to candidate performance. Our findings support the ability to successfully translate basic science courses for medical students in both traditional and online platforms. Although online teaching and learning in medical education is new, it has the potential to become mainstream in the future. With the ongoing global warming and climate change, there is more likelihood of future epidemics. In developing countries, governments with fewer resources can benefit from the cost-effectiveness that online education offers, thus, advancing access to medical education for more citizens in the general population. The COVID-19 pandemic has forced educators to examine elements of our medical education program. We now have an opportunity to sustain the adaptations to medical education during the pandemic by formulating a meticulous hybrid learning model that involves the integration of both online and traditional learning models in undergraduate medical education, and in that way, getting the “best of both worlds.”
